# Application of near-infrared spectroscopy technology in the complex fermentation system to achieve high-efficiency production

**DOI:** 10.1186/s40643-021-00452-9

**Published:** 2021-10-05

**Authors:** Chen Yang, Chen Lingli, Guo Meijin, Li Xu, Liu jinsong, Liu Xiaofeng, Chen Zhongbing, Tian Xiaojun, Zheng Haoyue, Tian Xiwei, Chu Ju, Zhuang Yingping

**Affiliations:** 1 State Key Laboratory of Bioreactor Engineering, East China University of Science and Technology, 130 Meilong Road, P.O. box 329, Shanghai, 200237 People’s Republic of China; 2 SDIC Biotech Investment Co. Ltd, Beijing, 100000 China; 3 SDIC Bioenergy Co. Ltd, Liaoning, 110000 China; 4 Zhejiang Biok Co.Ltd, Zhejiang, 310000 China; 5 Frontiers Science Center for Materiobiology and Dynamic Chemistry, East China University of Science and Technology, Shanghai, 200237 China

**Keywords:** Near-infrared spectroscopy, On-line, Lactic acid, Sophorolipids, Sodium gluconate

## Abstract

The fermentation process is dynamically changing, and the metabolic status can be grasped through real-time monitoring of environmental parameters. In this study, a real-time and on-line monitoring experiment platform for substrates and products detection was developed based on non-contact type near-infrared (NIR) spectroscopy technology. The prediction models for monitoring the fermentation process of lactic acid, sophorolipids (SLs) and sodium gluconate (SG) were established based on partial least-squares regression and internal cross-validation methods. Through fermentation verification, the accuracy and precision of the NIR model for the complex fermentation environments, different rheological properties (uniform system and multi-phase inhomogeneous system) and different parameter types (substrate, product and nutrients) have good applicability, and *R*^2^ was greater than 0.98, exhibiting a good linear relationship. The root mean square error of prediction shows that the model has high credibility. Through the control of appropriate glucose concentration in SG fermentation as well as glucose and oil concentrations SLs fermentation by NIR model, the titers of SG and SLs were increased to 11.8% and 26.8%, respectively. Although high cost of NIR spectrometer is a key issue for its wide application in an industrial scale. This work provides a basis for the application of NIR spectroscopy in complex fermentation systems.

**Supplementary Information:**

The online version contains supplementary material available at 10.1186/s40643-021-00452-9.

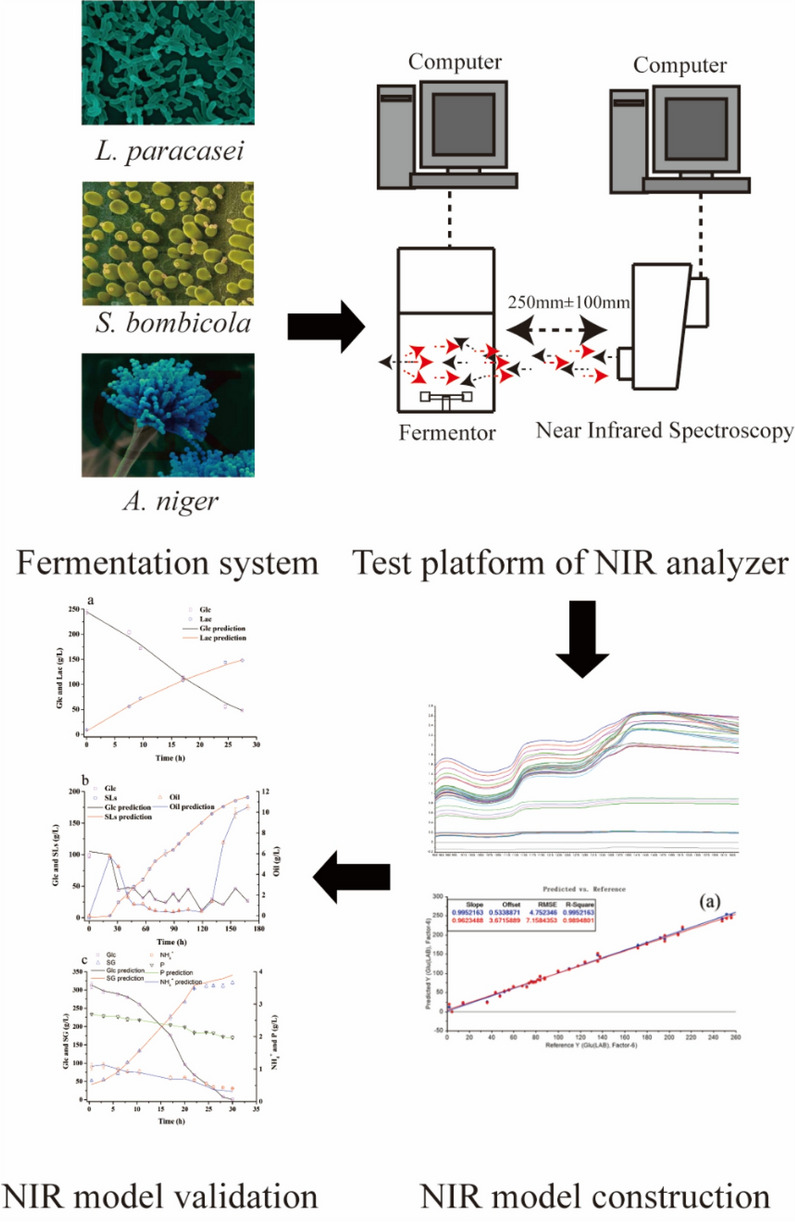

## Introduction

Fermentation is a dynamic and complex biochemical reaction process. Through real-time control of environmental parameters, the metabolic state of the cells can be regulated flexibly to obtain high titer, productivity and yield (Cai et al. 2002; Tian et al. 2018b; Wang 2019). Thus, bioprocess parameter detection is the basis of fermentation optimization (Zhang et al. 2004). With the development of sensing technology, on-line measurements of many physical, chemical and physiological parameters have been implemented (Zhang et al. 2014). However, more importantly, for key index parameters such as substrate and product concentrations in the broth, they are commonly determined by off-line methods, which is time-consuming and laborious from sampling to analysis procedures (Lorena et al. 2015).

Near-infrared (NIR) spectroscopy belongs to the molecular vibration spectrum, which is the fundamental frequency of molecular vibration frequency doubling and the combination of frequency for the characteristic information of hydrogen groups with X–H bond (X for C, O, N, S, etc.) (Costa et al. 2019; Peng et al. 2019). NIR spectroscopy is an accurate, on-line, and non-invasive technique, which does not require complicated sample preparation. Therefore, it has been widely applied to the quantitative and qualitative analyses in food, medicine and other fields (Quintelas et al. 2018; Pinto et al. 2015). Generally, NIR detection system consists of three parts with hardware equipment, stoichiometric software and mathematic model. According to the collected different spectral information, a quantitative functional relationship between spectral information and sample composition as well as sample content can be established (Wang et al. 2019). In the previous study, the application of NIR spectroscopy in biological process could be divided into three kinds according to sampling methods: off-line, at-line and on-line determination (Scarff et al. 2007). According to whether the near-infrared probe is in direct contact with the fermentation broth, it can be divided into contact type and non-contact type (Navrátil et al. 2005; Olarewaju et al. 2019; Svendsen et al. 2016). Do Nascimento et al. (2017) used near-infrared spectroscopy to detect ethanol, glucose, biomass and glycerol in the process of ethanol fermentation, and then used stoichiometry to associate the spectrals with off-line detecting data, and finally realized real-time and on-line detection in the ethanol fermentation process.

At present, the application of NIR spectroscopy combined with stoichiometry has been widely used in the fermentation processes. The difference of fermentation environment has a great impact on the NIR detection. Cervera et al. (2010) analyzed the application of NIR spectroscopy in the process of cell growth and fermentation, and found that structural differences (agitator paddle, baffle) in the bioreactor would lead to non-uniform bubble size and distribution in the fermentation broth. Otherwise, the morphology, substrates and products also affect the rheological properties (viscosity, color) of the fermentation broth, thus influencing the spectral absorption. Although NIR detection may work well in a homogeneous environment, complex fermentation systems seem to be a big challenge. In the fermentation process of sophorolipids (SLs), oil, solid particles and bubbles are mixed in the broth, in which three phases of gas, liquid and solid are present, making parameter detection very difficult. The fermentation of filamentous fungi is characterized by adherence, clump formation, breakage of mycelium, which exert a great influence on the rheological properties of fermentation broth, further on substrate and product detection. Therefore, the research on the application of NIR spectroscopy in different fermentation environments plays an important role in expanding its applicable range.

In this study, an experimental platform for real-time and on-line monitoring of the concentrations of substrates and products by NIR spectroscopy technology was developed in the fermentation processes of L-lactic acid (L-LA) by Lactobacillus paracasei, sodium gluconate (SG) by Aspergilus niger and SLs by Candida bombicola. Subsequently, a quantitative analysis model based on partial least-squares regression (PLSR) and internal cross-validation methods was established for spectral data collected in the fermentation processes. Finally, the feasible application of NIR spectroscopy technology in fermentation broths with different types of microbial strains and different rheological properties was verified.

## Materials and methods

### Strains, media and culture conditions

Lactobacillus paracasei NCBIO01 preserved by National Center of Bio-Engineering and Technology (NCBIO, Shanghai) was used to produce L-LA. Fermentation medium contained (g/L): glucose 250, yeast extract 13.33, peptone 13.33, beef extract 13.33, sodium acetate anhydrous 0.67, NaCl 0.0133, FeSO_4_ 0.0133, MnSO_4_ 0.0133, MgSO_4_·7H_2_O 0.0133. The L-LA fermentation was carried out in a 5-L ordinary glass bioreactor (Shanghai Guoqiang Bioengineering Equipment Co., Ltd., China) with 4 L initial working volume. The operation conditions were as follows: inoculum of 20% (v/v), fermentation temperature of 37 ℃, aeration of 0.025 vvm, agitation of 150 rpm. The supply of different oxygen levels was accomplished by adjusting the agitation. The process pH was maintained at 6.0 by adding 25% NH_4_OH solution.

Lactobacillus paracasei NCBIO01 preserved by National Center of Bio-Engineering and Technology (NCBIO, Shanghai) was used to produce L-LA. Fermentation medium contained (g/L): glucose 250, yeast extract 13.33, peptone 13.33, beef extract 13.33, sodium acetate anhydrous 0.67, NaCl 0.0133, FeSO4 0.0133, MnSO4 0.0133, MgSO4·7H2O 0.0133. The L-LA fermentation was carried out in a 5-L ordinary glass bioreactor (Shanghai Guoqiang Bioengineering Equipment Co., Ltd., China) with 4 L initial working volume. The operation conditions were as follows: inoculum of 20% (v/v), fermentation temperature of 37 ℃, aeration of 0.025 vvm, agitation of 150 rpm. The supply of different oxygen levels was accomplished by adjusting the agitation. The process pH was maintained at 6.0 by adding 25% NH4OH solution.

Aspergilus niger which was kindly provided by Shangdong Fu Yang Biotechnology Co., Ltd was used to produce sodium gluconate (SG). The initial fermentation medium contained (g/L): glucose 250, KH_2_PO_4_ 0.5, (NH_4_)_2_SO_4_ 2.355, (NH_4_)_2_HPO_4_O 1.8, corn steep liquor 10. The SG fermentation was carried out in a 5-L bioreactor with 3 L initial working volume. The operation conditions were as follows: inoculum of 10%, fermentation temperature of 38℃, aeration of 1.2 vvm, agitation of 500–700 rpm. The process pH was maintained at 5.3 by adding 7.5 M NaOH solution.

Candida bombicola ATCC 22214 bought from Guangdong Culture Collection Center (China) was used to produce SLs and it was stored at − 80 ℃ in 20% glycerol solution. The initial fermentation medium contained (g/L): glucose 100, KH_2_PO_4_ 1, (NH_4_)_2_SO_4_ 4, MgSO_4_·7H_2_O 0.5, corn steep liquor 10. The SLs fermentation was carried out in a 5-L bioreactor with 2.5 L initial working volume. The operation conditions were as follows, inoculum of 2.9% (optical density of 80 at 600 nm), fermentation temperature of 25℃, aeration of 0.5 vvm, initial agitation of 200 rpm. The process pH was maintained at 3.5 by adding 4 M NaOH solution. The dissolved oxygen (DO) was controlled above 40–25% by adjusting the agitation step-wisely. The rapeseed oil was continuously fed into the broth, maintaining the level lower than 10 g/L. The glucose was added per 12 h to keep the level between 30 and 50 g/L.

### Analytical methods

#### Off-line determination of glucose and L-LA in lactic acid fermentation

L-LA and glucose were measured by SBA-40C Biosensor analyzer (Shandong Province Academy of Sciences, China).

#### Off-line determination of glucose, SG, NH_4_^+^ and soluble phosphorus concentrations in SG fermentation

Glucose concentration in the broth was analyzed by the method as above mentioned. SG concentration in the broth was determined by HPLC (SPD-20A, Shimadzu, Japan) at 210 nm with a C_18_ column (4.6 × 250 nm, no. 336–1101, Sepax Technologies, Inc., USA) as described by Tian et al. (2018a). The mobile phase was prepared with isovolumetric methanol solution (3 M) and phosphate solution (0.25 M). The flow rate and the column temperatures were set at 1.0 mL/min and 28 ℃, respectively.

Soluble phosphorus (P) concentration in the broth was determined by ammonium molybdate reduction method at 825 nm, as described by Durge and Paliwal (1967). NH_4_^+^ was determined by phenol–sodium hypochlorite colorimetry at 625 nm, as described by Broderick and Kang (1980).

#### Off-line determination of glucose, oil and SLs concentrations in SLs fermentation

Glucose concentration in the broth was analyzed by the method as above mentioned. Oil and SLs concentrations were determined by the weighing method and high-performance liquid chromatography (HPLC) method, respectively, as described in our previous works (Chen et al. 2019b). Briefly, three parallel broth samples were extracted twice using the same volume of n-hexane for oil content determination. The upper layer was then transferred to another tube and dried for 24 h to constant weight by an oven. In terms of SLs, two milliliter of fermentation broth was withdrawn and 2 mL of KOH/MeOH (4 M) solution was added, and then the mixture was heated at 80 ℃ for 15 min. After cooling to room temperature, methanol was added to a total volume of 10 mL and NaH_2_PO_4_ pH buffer (0.2 M) was used to neutralize the solution. Finally, the sample was diluted to an appropriate concentration for HPLC analysis. Mobile phase (ammonium acetate, 0.02 mol/L; formic acid, 1% v/v; methanol, 75% v/v) C_18_ column (4.6 mm × 250 mm, Acchrom) refractive index detector (RID), and the flow rate of 0.9 mL/min were adopted. The injection volume was 20 μL and the column and detector temperatures were controlled at 50 ℃ and 35 ℃, respectively.

### On-line determination of substrate and product concentrations by NIR method

#### NIR spectroscopy acquisition and analysis

**Fig. 1:**
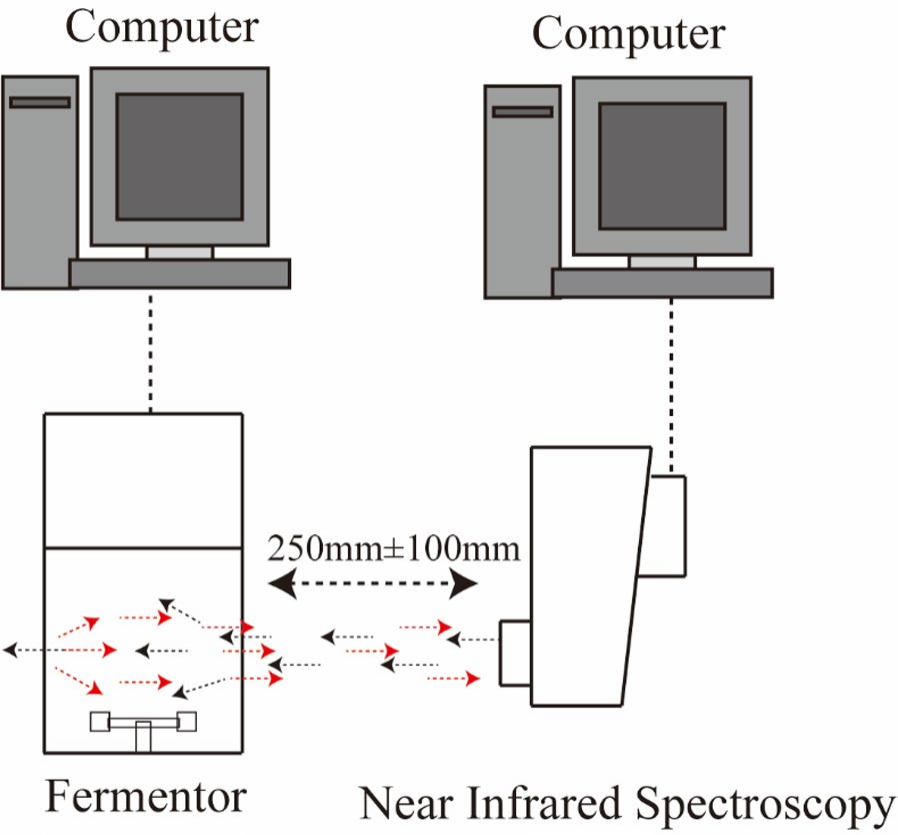
Test platform based on NIR analyzer for fermentation process parameters

DA7440 on-line NIR analyzer manufactured by Perten company (Sweden) was used in fermentation processes. Light emitted from the instrument, passed through the glass jar, and after many reflections or refraction, the device reabsorbs the returned spectrum (Fig. 1). It belongs to non-invasive diffuse reflection detector and an unscrambler 10.3 quantitative analysis software from NIR spectroscopy can conduct spectral preprocessing. Spectral region was selected as 900–1650 nm (removing the interference of water vapor from the wavelength range of 1350–1410 nm) and remove abnormal samples. The spectral acquisition speed was about 30 times full spectrum measurements every second. All received spectral signal results were stored and displayed on a connected computer.

#### NIR spectral modeling

In order to eliminate the interference from the changes of environmental conditions on the spectrum measurement, the methods such as first-order derivative, five-point smoothing, standard normal variable (SNV), de-trending algorithm were adopted to preprocess spectral data so as to improve the detection accuracy and reliability in this study. In the early stage of the study, PLSR, multivariate linear regression (MLR), principal component regression (PCR) and support vector machine regression (SVMR) were compared (20 samples of SLs fermentation). The comparison of the prediction results of each model shows that PLSR has better prediction effect (Additional file 1: Table S1).

PLSR is a new kind of multivariate statistical data analysis method (Rehman et al. 2018). PLSR is a combination of principal component analysis (PCA), canonical correlation analysis (CCA) and multiple linear regression analysis (Chen et al. 2019a). The mathematical model was as follows by Eqs. (1) and (2):

1 $${\text{X}} = {\text{TP}} + {\text{E}},$$

2 $${\text{Y = UQ + F,}}$$

where the matrix X and Y represent the independent (absorbance matrix) and dependent (concentration matrix) variable matrixes, respectively. The matrix T and U are the score matrixes of X and Y, respectively. The matrix P and Q represent the load matrixes for X and Y. E and F represent the errors. PLSR decomposed the spectral matrix and the concentration matrix at the same time, and considered the relationship between them during the decomposition to strengthen the corresponding machine damage, so as to ensure the best correction model.

Cross-validation (CV) is a commonly used statistical method, which often cuts data samples into smaller subsets. In a given modeling sample, most samples are taken for modeling and a small number of samples are left for evaluation by the established model (Ryan et al. 2013). In this study, the internal cross-validation method was used to determine the optimal factor number in the mathematical model (Afendras and Markatou 2019). The external validation procedure consists of using validation samples that do not belong to the prediction set.

#### NIR model evaluation

In order to evaluate the prediction function of NIR spectroscopy for fermentation process parameters, the root mean square error of prediction (RMSEP) and correlation coefficient *R*^2^ of the prediction set were adopted. The calculation formulas are as follows by Eqs. (3) and (4) (Rodrigues et al. 2008; Dong et al. 2018):

3 $${\text{RMSEP}} = \sqrt {\frac{1}{n}\sum\nolimits_{{i{ = 1}}}^{n} \left( {{\text{y}}_{i} - \widehat{{{\text{y}}_{{1}} }}} \right)^{2} ,}$$

4 $$R^{2} = 1 - \frac{{\sum_{{i{ = 1}}}^{n} \left( {{\text{y}}_{i} - \widehat{{{\text{y}}_{{1}} }}} \right)^{{2}}  }}{{\sum _{{i{ = 1}}}^{n} \left( {{\text{y}}_{i} - \widehat{{{\text{y}}_{m} }}} \right)^{{2}}  }},$$

where y_*i*_ represents the reference value of sample *i*, $\widehat{{\mathrm{y}}_{\mathrm{i}}}$ represents predicted value of sample *i*, and y_*m*_ represents the sample mean to be predicted. RMSEP was used to evaluate the deviation between the predicted value and the reference value. *R*^2^ represents the correlation between the predicted value and the reference value. The smaller the RMSEP, the larger is *R*^2^, indicating the higher accuracy of the NIR model.

### Statistical analysis

All experiments were performed in triplicate and all data were presented as the mean with standard deviation (SD). Statistical analysis was performed using one-way analysis of variance (ANOVA) and *t* test (*P* < 0.05) was used to test whether there was any significant difference among treatments (SPSS 22.0, SPSS Inc., USA).

## Results

### Quantitative calibration models by NIR method

**Fig. 2:**
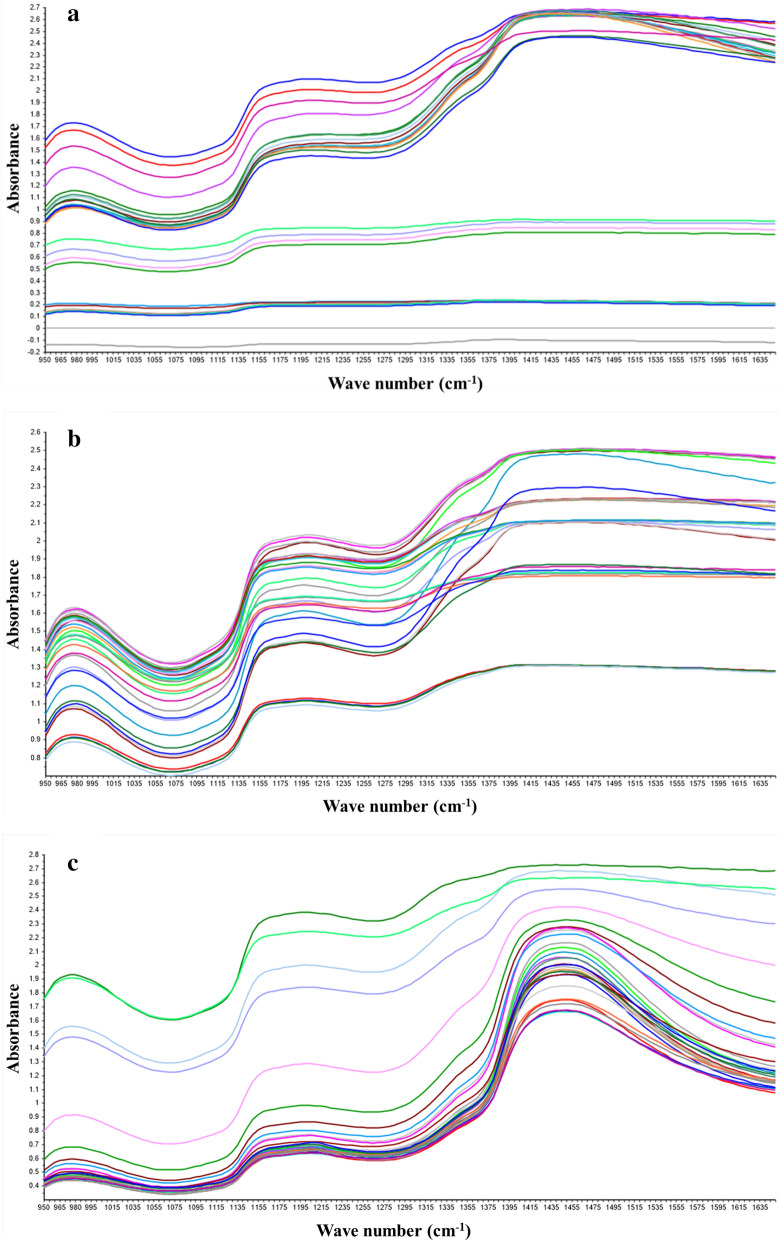
NIR absorption spectra for fermentation broth. **a** L-LA fermentation broth; **b** SG fermentation broth; **c** SLs fermentation broth. The line represents the absorbance of fermentation broth at different wave numbers, and the color represents different time points

**Fig. 3:**
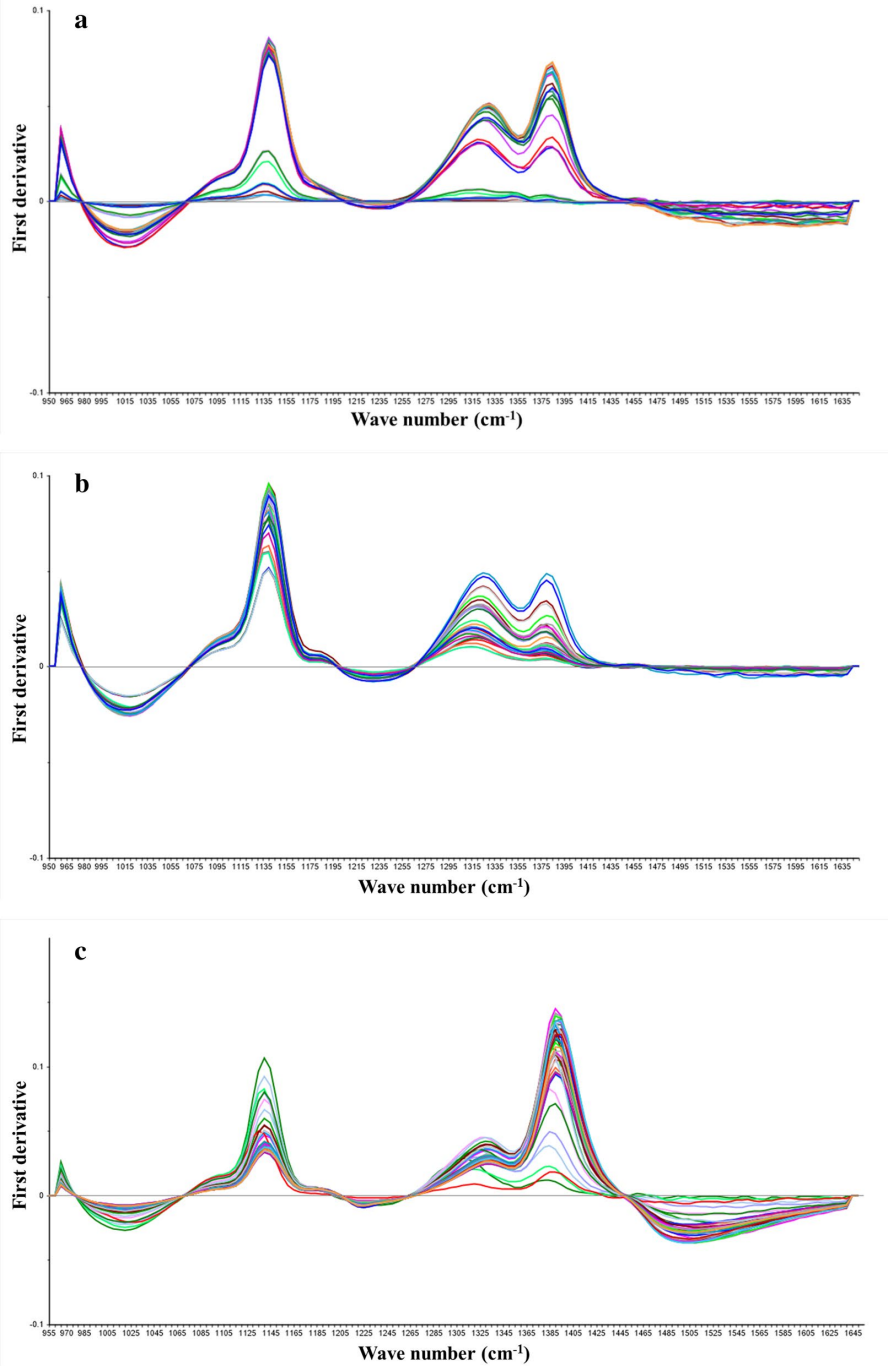
NIR spectra of samples using first derivative. **a** L-LA fermentation; **b** SG fermentation; **c** SLs fermentation

**Fig. 4:**
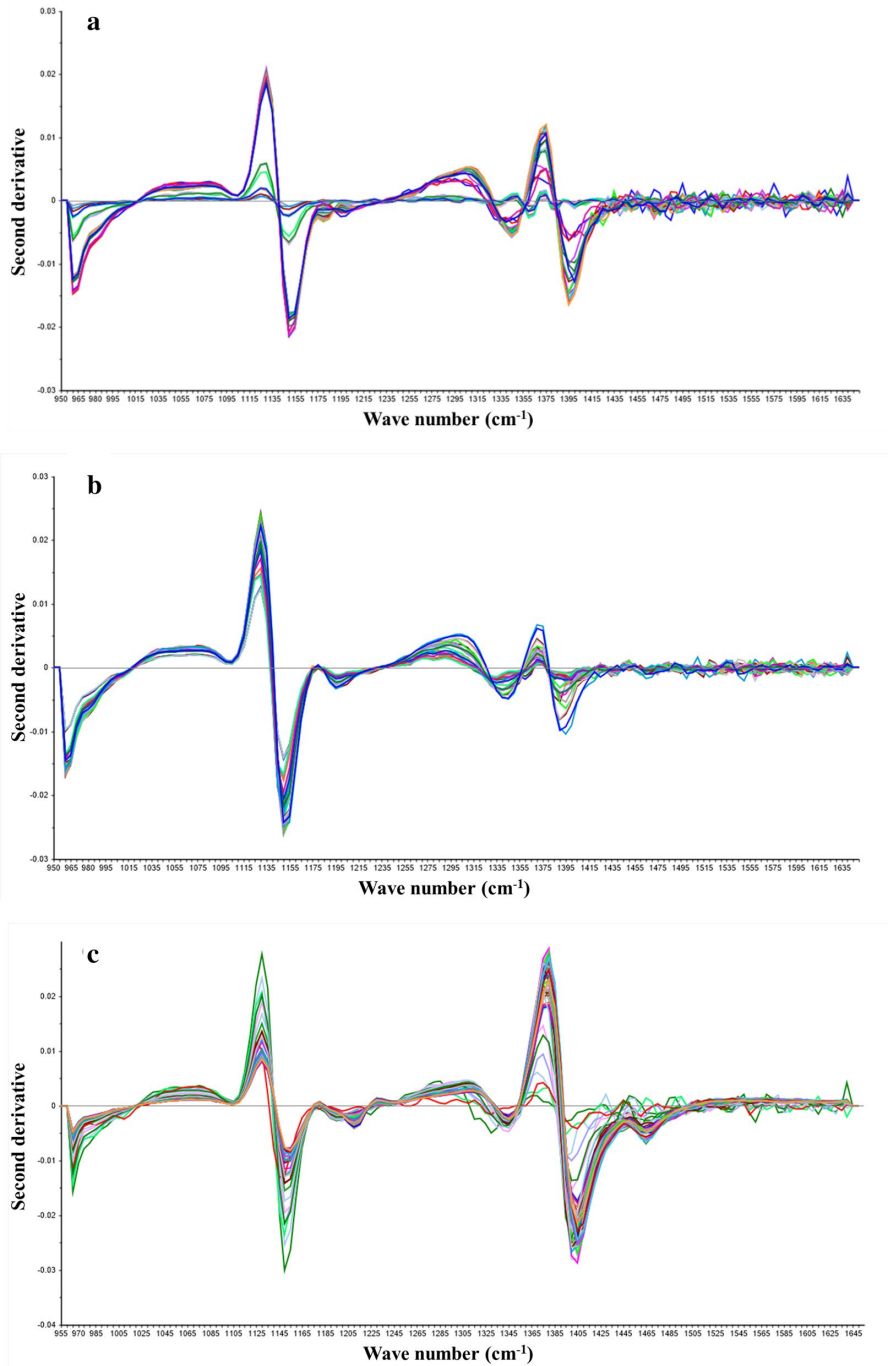
NIR spectra of samples using second derivative. **a** L-LA fermentation; **b** SG fermentation; **c** SLs fermentation

The data of the L-LA, SLs and SG fermentation parameters were collected on the above NIR experimental platform to establish the calibration model of NIR spectrum. The off-line sampling data of glucose, L-LA, SLs, rapeseed oil, SG, NH_4_^+^and P are shown in Table 1. Samples collected used as prediction set and calibration set were 100 and 30, respectively (Table 1). In order to ensure the reliability of the spectral model, the maximum and minimum values of the off-line detection data in the fermentation process were included in the prediction set. The differences of substrates or products in the fermentation process will cause significant changes in the spectral data (Fig. 2a, b, c). In addition, compared with L-LA fermentation, SG and SLs fermentation broths present the characteristics of multi-phase, viscosity change and mycelium interference, therefore the spectral baseline drift was relatively large. To enhance spectral features, the original spectra need to be subjected to pretreatments before being used to construct calibration models. The first and second derivatives can eliminate the baseline drift related to the changes in concentrations, as shown in Fig. 3a, b, c, and Fig. 4a, b, c were the spectra data processed by the first and second derivatives. Regression coefficients were established according to the off-line data and spectral data of each fermentation component, which indicated that the absorption signal intensity of different components in fermentation broth was different at wavelength (Additional file 1: Fig S1–S9). It was noted that the spectra do not define a substance in a clear way, but rather represents the absorbance of different groups of a substance at the wavelength.

**Table 1:** Parameters of fermentation process

Fermentation process	Component	Prediction set (calibration set)	Calibration range (g/L)
LA fermentation	Glucose	102 (31)	2–256
	L-LA	102 (31)	6.3–156
SG fermentation	Glucose	103 (32)	2–312
	SG	103 (32)	49.33–359.8
	NH_4_^+^	103 (32)	0.38–1.93
	P	103 (32)	1.97–4.33
SLs fermentation	Glucose	105 (35)	8.34–110.96
	SLs	105 (35)	3.59–151.97
	Residual oil	105 (35)	0.57–29.05

**Fig. 5:**
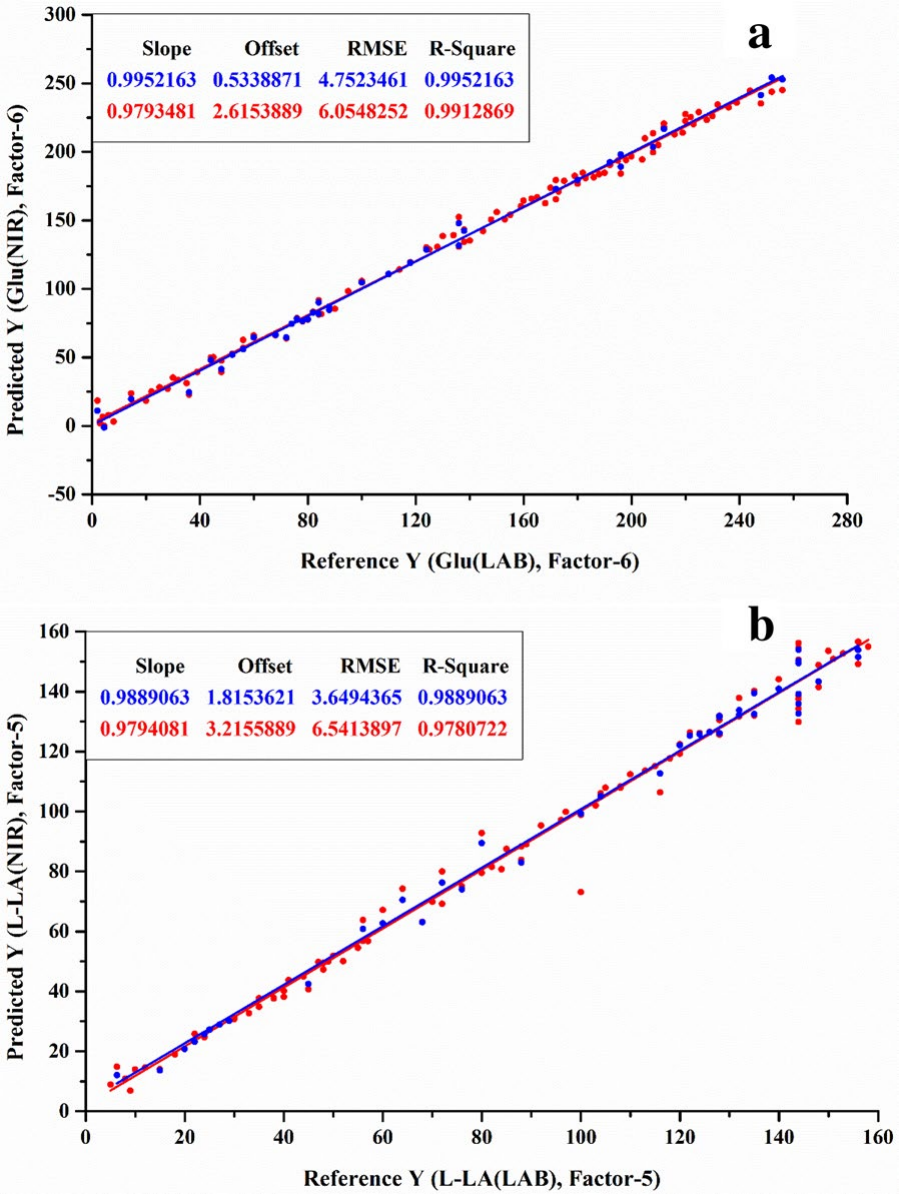
Spectral model of L-LA fermentation **a** glucose, **b** L-LA. The X-axis is the reference value for off-line detection and the Y-axis is the predicted value based on NIR data. The blue color in figure represents the calibration set data obtained by the CV method, and the red color in figure represents the prediction set

**Fig. 6:**
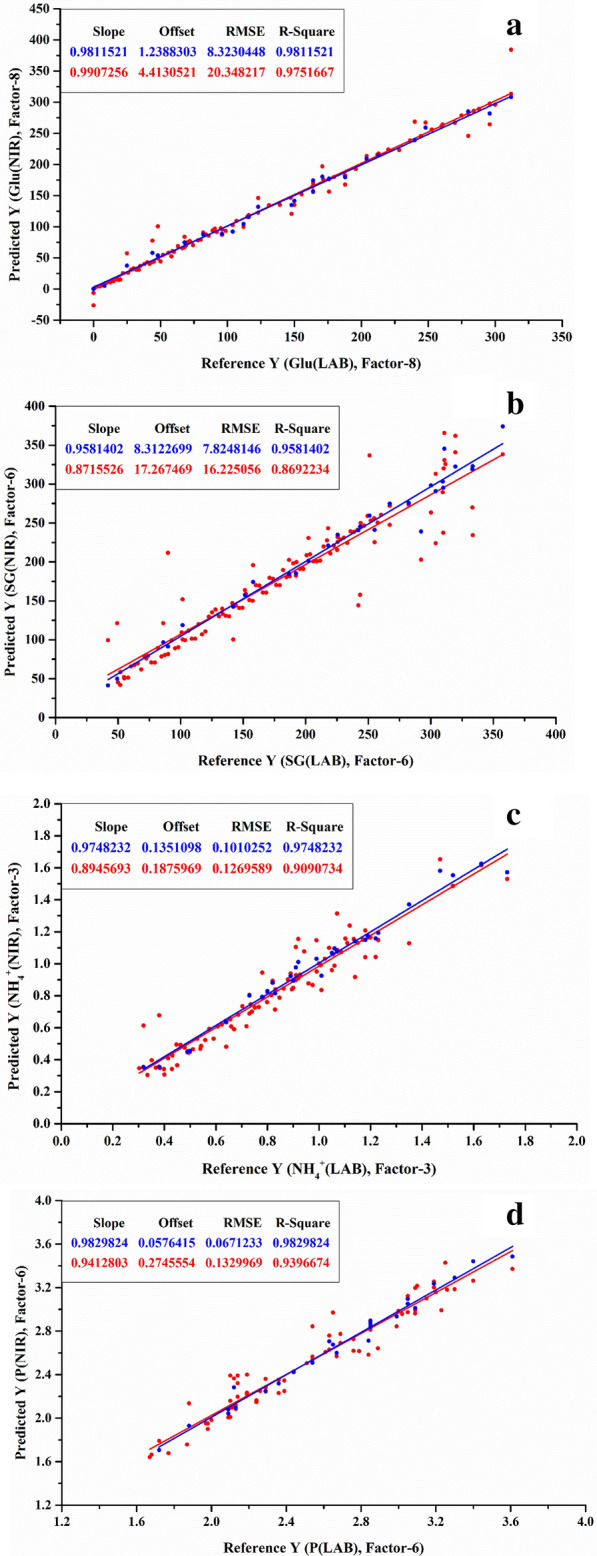
Spectral model of SG fermentation **a** glucose, **b** SG, **c** NH_4_^+^, **d** P

**Fig. 7:**
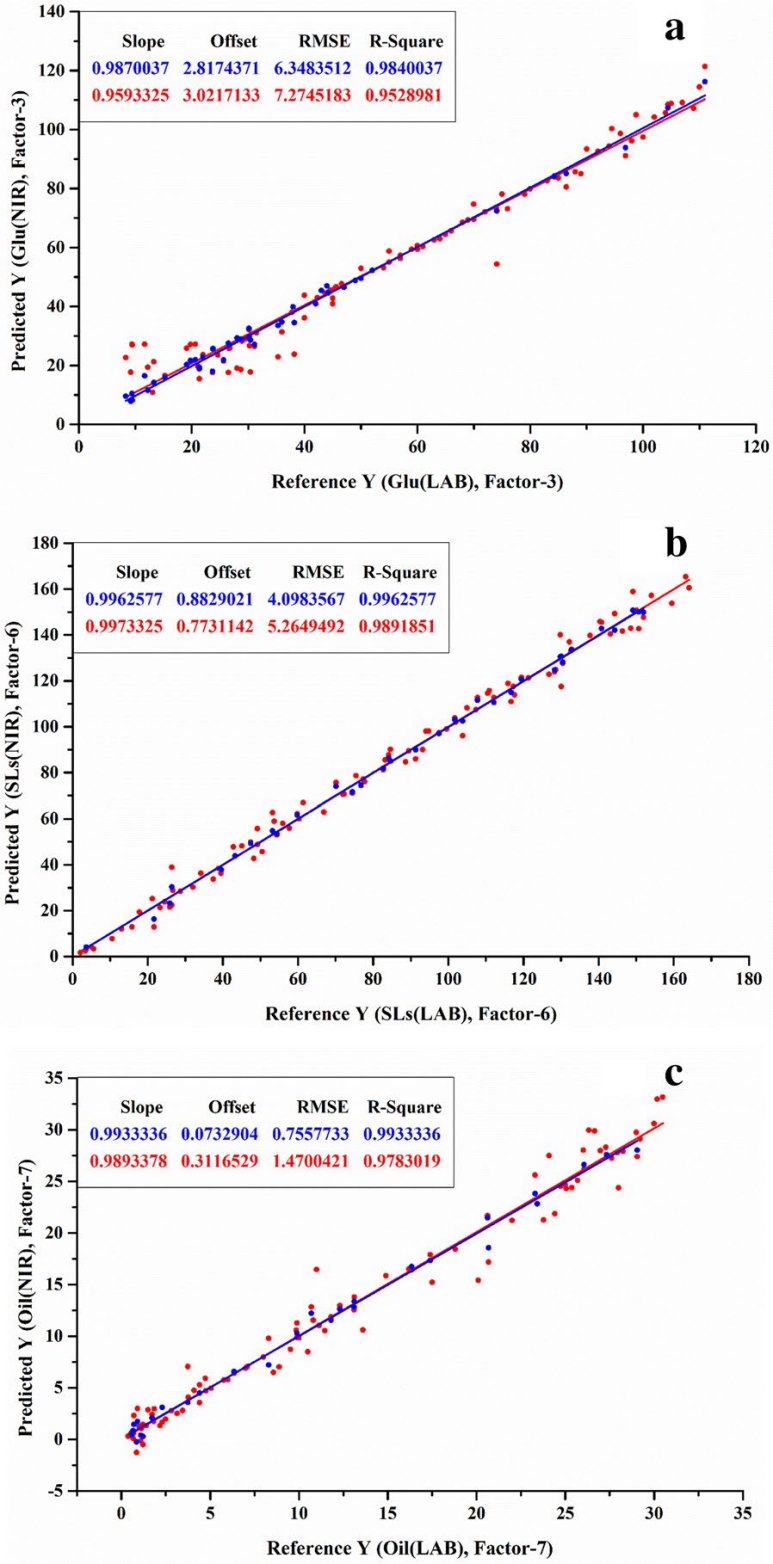
Spectral model of SLs fermentation: **a** glucose, **b** SLs, **c** residual oil

The spectral calibration model was established by PLSR and CV methods, and then the model was validated by the known validation data set (Table 2). L-LA fermentation was relatively simple and the NIR model had a good prediction function on environmental glucose and L-LA concentrations with both *R*^2^ above 0.989 (Fig. 5a and b). With regard to SG concentration (*R*^2^=0.958), the prediction effects of glucose, NH_4_^+^ and P contents were better, where *R*^2^ were 0.981, 0.975 and 0.983 in the SG fermentation process, respectively (Fig. 6a, b, c and d). In terms of SG, it could contribute to the significant change of SG concentration during the fermentation process. On the other hand, compared to SLs fermentation, the NIR model also could well predict environmental SLs and residual oil contents with *R*^2^ over 0.996 and 0.993, respectively (Fig. 7b and c), however, it was noteworthy that the *R*^2^ of glucose concentration only reached 0.984 (Fig. 7a), which might be ascribed to the intermittent addition of solid glucose into the broth during the process, leading to a wide fluctuation of glucose concentration in a relatively short period.

**Table 2:** Spectral prediction model performance index

	L-LA fermentation		SG fermentation				SLs fermentation		
	Glu	L-LA	Glu	SG	NH_4_^+^	P	Glu	SLs	Oil
RMSEP	4.752	3.650	8.323	7.825	0.101	0.0671	6.348	4.098	0.756
*R*^2^	0.995	0.989	0.981	0.958	0.975	0.983	0.984	0.996	0.993

### NIR model validation

**Fig. 8:**
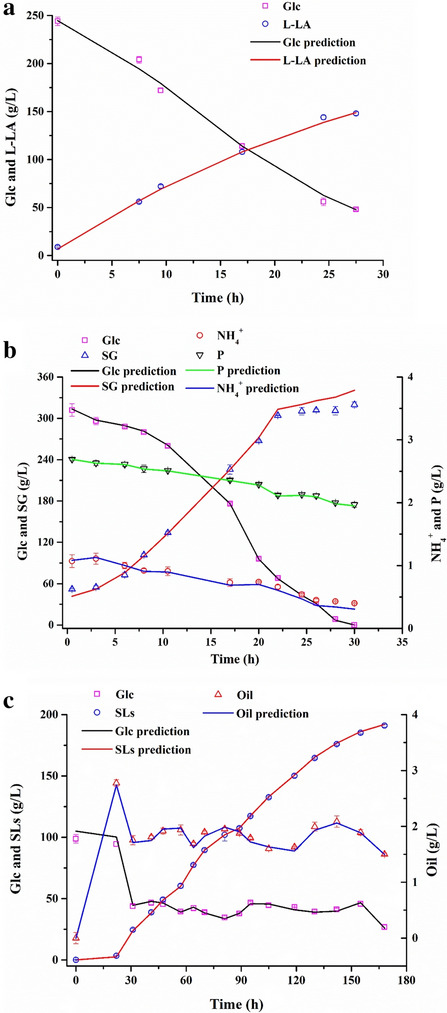
Comparison of on-line and off-line detection of fermentation process parameters. **a** L-LA fermentation, **b** SG fermentation, **c** SLs fermentation

Three independent batches of L-LA, SG and SLs fermentation were conducted, respectively, to validate the above established NIR models. As shown in Fig. 8, the line data were the real-time predicted values based on the NIR model and the scatter data were the reference values by off-line detection (Fig. 8a, b and c). It was found that the real-time on-line detection results of the NIR model have a high correlation with the reference values of the off-line detection (Additional file 1: Table S2). Apart from the SG of 0.975, all the *R*^2^ of other components were above 0.980, showing a good correlation (Fig. 8). On the other hand, compared with RMSEP in the process of model establishment, the reduction of RMSEP means that the error was reduced and the model was more accurate during the verification process.

### NIR model application

**Fig. 9:**
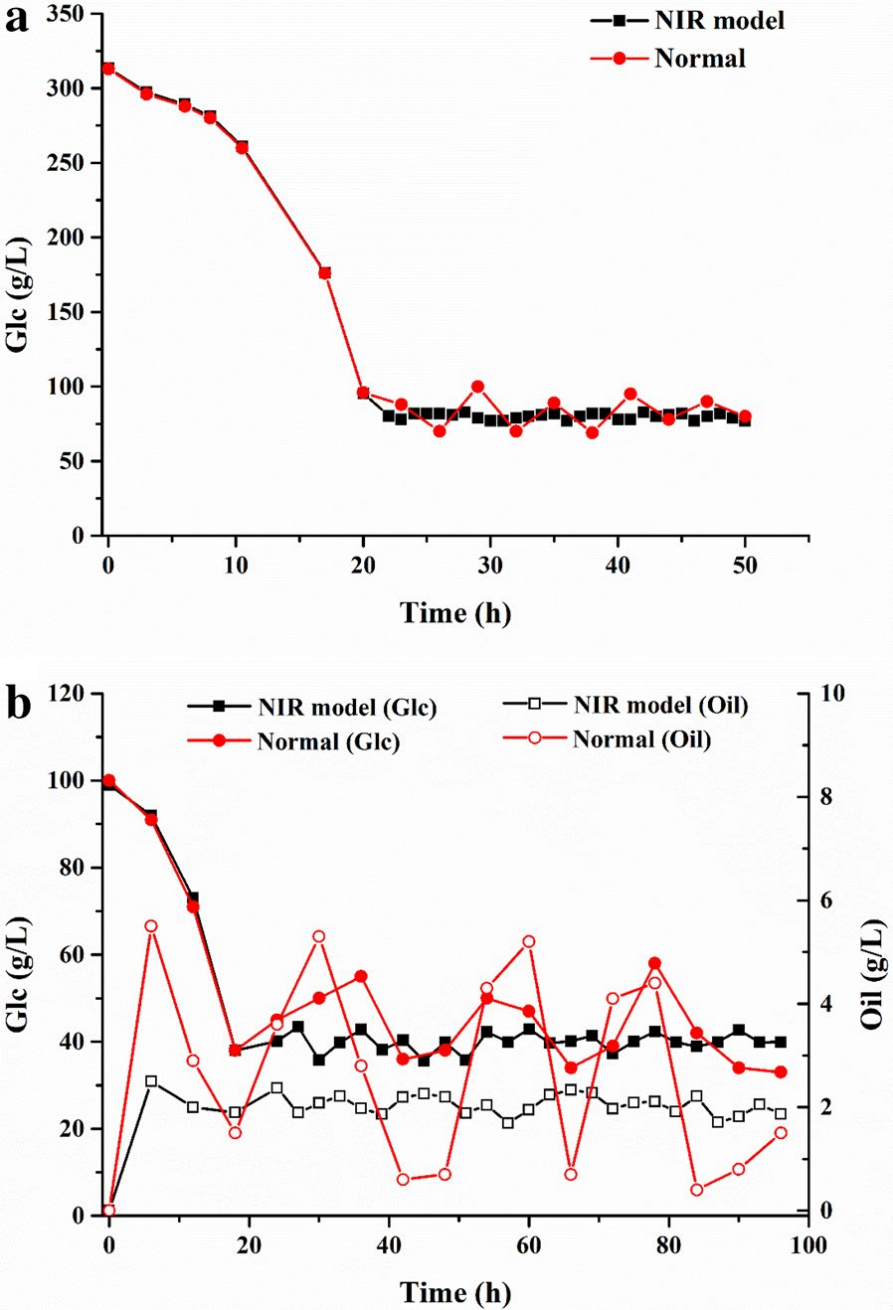
Application of NIR model for **a** glucose concentration regulation in SG fermentation; **b** glucose and oil concentrations regulation in SLs fermentation

In the process of SG fermentation, high concentration of glucose (> 50 g/L) would inhibit the synthesis of secondary metabolites (yellow pigments, which will affect the quality of the product). Herein, the glucose concentration was maintained at 80.1 g/L, with an error of 2.0 g/L by the NIR model to improve the quality of SG (Fig. 9a). In contrast, the average residual glucose concentration was just controlled at 82.9 g/L, with an error of up to 11.1 g/L by conventional pulse feed model. In addition, the NIR model could shorten the control period from 3 to 1 h. On the other hand, in the SLs fermentation, the NIR spectroscopy platform could realize real-time monitoring of the residual oil and glucose concentrations in the fermentation broth, and then their concentrations were controlled at about 2.1 g/L and 40.3 g/L, respectively (Fig. 9b). Similarly, the control period was shortened from 6 to 3 h, and the control errors of rapeseed oil and glucose were reduced from 1.9 g/L and 8.4 g/L to 0.3 g/L and 2.8 g/L, respectively. Consequently, it was found that the titers of SG and SLs increased to 11.8% and 26.8%, respectively (Table 3). This result further indicates that the NIR model has a good prediction accuracy for the detection of fermentation process parameters.

**Table 3:** Comparison of NIR model and normal regulation

Model	SG titer (g/L)	SLs (g/L)
Normal	470.25	150.56
NIR model	525.61	190.84

## Discussion

Fermentation process was characterized of complexity and uncertainty, and the basis of fermentation regulation is to harness fermentation parameters in real-time. However, at present, the detection of process parameters is still relatively limited, and it is impossible to achieve multi-dimensional cell metabolism measurement. Moreover, some sensors may be affected by the characteristics of strain, the rheological properties of fermentation broth, the aseptic requirement, so as the on-line detection of various substances and products in the fermentation broth cannot be realized. At present, a variety of on-line detection technologies have been used in microbial fermentation, including conventional environmental sensors (DO, pH and temperature electrode), exhaust gas mass spectrometry, infrared spectroscopy, low field nuclear magnetic resonance, Raman spectroscopy, viable cell sensor, electronic nose (Zhao et al. 2016; Schalk et al. 2019; Wang et al. 2016; Feng et al. 2021). Do Nascimento et al. (2017) applied NIR spectroscopy to realize real-time and on-line detection of ethanol, glucose, biomass and glycerol in the process of ethanol fermentation. Puvendran et al. (2018) used NIR spectroscopy technology to monitor the fermentation process parameters of hyaluronic acid in real-time, and then an established quantitative analysis model with partial least-squares regression method was applied to other hyaluronic acid-producing processes by the recombinant strains. Wang et al. (2020) established a method to quickly identify the quality of Japanese fermented soy sauce based on NIR spectroscopy technology and chemometric method, which could realize rapid and economical classification of soy sauce. Although near-infrared spectroscopy technology has relatively mature applications in simple fermentation systems (Sandor et al. 2013; Bence et al. 2019), in view of the complex fermentation system, especially in the complex environment of SLs fermentation broth with gas–liquid–solid three-phase, the application of NIR spectroscopy technology has not been ever reported. Traditional HPLC and organic solvent extraction methods are adopted for determining the concentrations of residual oil and SLs, which is time- and labor-consuming, as well as requires the use of organic solvents. Chen et al. (2019b) realized real-time detection of SLs and oil in fermentation broth with low field nuclear magnetism, which greatly shortened the detection time, however the pre-treatment process was still indispensable. The application of NIR spectroscopy technology was used to monitor the residual glucose, residual oil and SLs concentrations in fermentation broth in real-time, which was of great significance for developing an efficient SLs production mode by semi-continuous fermentation. At present, most of NIR spectroscopy and Raman spectroscopy techniques use contact electrode probes. In the fermentation process, it is necessary to face high-temperature and high-pressure sterilization and fermentation liquid corrosion, which puts forward higher requirements on the electrode. During the fermentation of filamentous fungi, hyphae are inevitably formed, and the hyphae are easily wrapped on the electrode, which brings great challenges to the detection. In this study, the developed non-contact NIR spectroscopy technology could effectively overcome these difficulties. However, with the current on-line NIR spectroscopy technology, it may be difficult to achieve large-scale applications. The main reason is the high cost of NIR spectrometer as well as the anti-interference of the instrument against the harsh environments in the factory. Otherwise, it is believed that more advanced sensing technologies, such as Raman spectroscopy technology, exhaust gas mass spectrometry technology, viable cell detection technology, could be combined to achieve real-time and on-line detection of cellular metabolic characteristic parameters from multi-scale levels, laying a foundation for the intelligent control of the fermentation process in near future.

## Conclusion

A NIR detecting platform has been established to real-time and on-line detection of process multi-parameters under different fermentation systems. Especially for the complex fermentation environments, different rheological properties (uniform system and multi-phase inhomogeneous system) and different parameter types (substrate, product and nutrients) can also have good applicability. The verification shows that the NIR model has good predictability and reliability. In addition, the application of NIR model in the fermentation of SLs and SG was studied. It can be found that NIR model can achieve stable regulation for complex fermentation system, maintain a certain concentration of substrate glucose and rapeseed oil, and achieve titers of SG and SLs that increased to 11.8% and 26.8%, respectively. NIR model was helpful for the process control and provided a solid technical basis for the fermentation regulation.

### Supplementary Information

**Additional file 1:** **Table S1.** Comparison of different chemometrics. **Fig. S1.** Absorption signals of glucose in L-LA fermentation broth at NIR wavelengths. **Fig. S2.** Absorption signals of L-LA in L-LA fermentation broth at NIR wavelengths. **Fig. S3.** Absorption signals of glucose in SG fermentation broth at NIR wavelengths. **Fig. S4.** Absorption signals of SG in SG fermentation broth at NIR wavelengths. **Fig. S5.** Absorption signals of NH in SG fermentation broth at NIR wavelengths. **Fig. S6.** Absorption signals of NH4+ in SG fermentation broth at NIR wavelengths. **Fig. S7.** Absorption signals of glucose in SLs fermentation broth at NIR wavelengths. **Fig. S8.** Absorption signals of SLs in SLs fermentation broth at NIR wavelengths. **Fig. S9.** Absorption signals of oil in SLs fermentation broth at NIR wavelengths. **Table S2.** Spectral validation of model performance index.

## Data Availability

All data generated or analyzed during this study are included in this published article.
