# Supplementary material for: Application of near-infrared spectroscopy technology in the complex fermentation system to achieve high-efficiency production
Source: Bioresour Bioprocess. 2021 Oct 5;8(1):96. doi: 10.1186/s40643-021-00452-9 (PMC11368886; doi:10.1186/s40643-021-00452-9)
Supplement: Supplementary file 1 — Additional file 1: Table S1. Comparison of different chemometrics. Fig. S1. Absorption signals of glucose in L-LA fermentation broth at NIR wavelengths. Fig. S2. Absorption signals of L-LA in L-LA fermentation broth at NIR wavelengths. Fig. S3. Absorption signals of glucose in SG fermentation broth at NIR wavelengths. Fig. S4. Absorption signals of SG in SG fermentation broth at NIR wavelengths. Fig. S5. Absorption signals of NH in SG fermentation broth at NIR wavelengths. Fig. S6. Absorption signals of NH4+ in SG fermentation broth at NIR wavelengths. Fig. S7. Absorption signals of glucose in SLs fermentation broth at NIR wavelengths. Fig. S8. Absorption signals of SLs in SLs fermentation broth at NIR wavelengths. Fig. S9. Absorption signals of oil in SLs fermentation broth at NIR wavelengths. Table S2. Spectral validation of model performance index. [file 40643_2021_452_MOESM1_ESM.docx]

**Application of near infrared spectroscopy technology in the complex fermentation system to achieve high-efficiency production**

Chen Yang^a^, Chen Lingli^a^, Guo Meijin^a^, Li Xu^a*^, Zeng Wei^b^, Chen Zhongbing^c^, Tian Xiwei^a*^, Chu Ju^a^, Zhuang Yingping^a,d^

^a^ *State Key Laboratory of Bioreactor Engineering, East China University of Science and Technology, Shanghai 200237, China,*

^b^*Yidu HEC Biochem. Co. Ltd., Hubei 443300, PR China,*

^c^ *Zhejiang Biok Co.Ltd, Zhongguan Industrial Park, Zhejiang, PR China,*

^d^ Frontiers Science Center for Materiobiology and Dynamic Chemistry*, East China University of Science and Technology, Shanghai 200237, China*

**Author for correspondence*

*Address:*

*Xiwei Tian, Xu Li*

*State Key Laboratory of Bioreactor Engineering, East China University of Science and Technology*

*P.O. box 329, 130 Meilong Road, Shanghai 200237, People’s Republic of China*

*Tel:* *+86-21-64253021; Tel: +**86-21-64253853*

*E-mail address: tahfy@163.com (Xiwei Tian), xli@ecust.edu.cn (Xu L)*

**Supplementary materials**

*1. Methods contrast*

Table S1 Comparison of different chemometrics

| Methods | L-LA | | SG | | SLs | |
| --- | --- | --- | --- | --- | --- | --- |
|  | R^2^ | RMSEP | R^2^ | RMSEP | R^2^ | RMSEP |
| PLSR | 0.9887 | 5.2433 | 0.9812 | 8.7362 | 0.9832 | 7.2123 |
| MLR | 0.8635 | 8.3452 | 0.8973 | 10.2613 | 0.8563 | 9.5372 |
| PCR | 0.9124 | 6.4389 | 0.9273 | 9.9642 | 0.9365 | 8.9273 |
| SVMR | 0.9021 | 6.7153 | 0.9234 | 9.5812 | 0.9573 | 8.6512 |

*2. NIR absorption signals of different wavelengths*


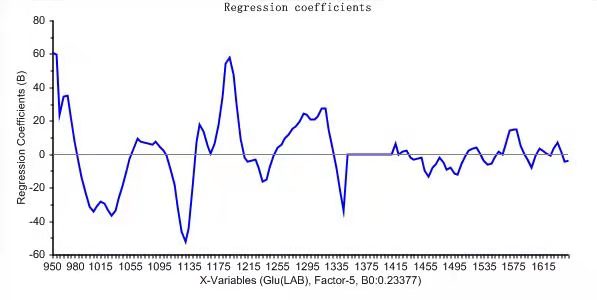


**Fig. S1.** Absorption signals of glucose in L-LA fermentation broth at NIR wavelengths


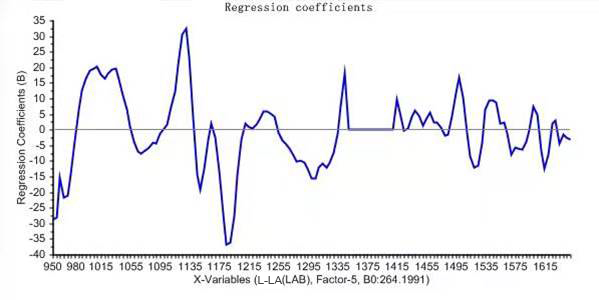


**Fig. S2.** Absorption signals of L-LA in L-LA fermentation broth at NIR wavelengths


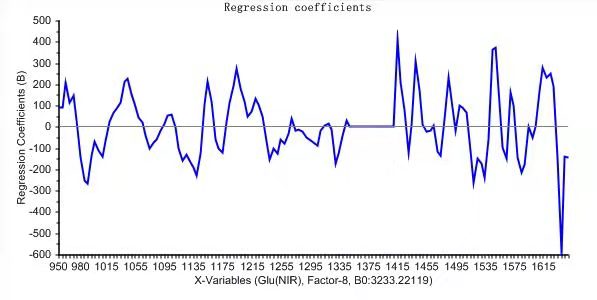


**Fig. S3.** Absorption signals of glucose in SG fermentation broth at NIR wavelengths


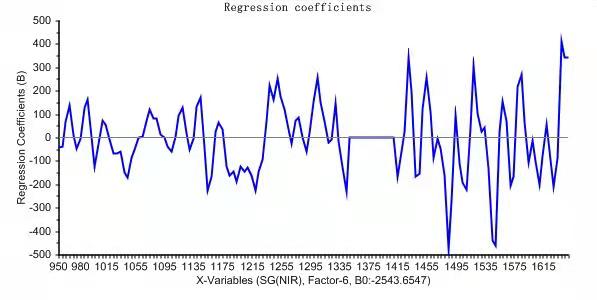


**Fig. S4.** Absorption signals of SG in SG fermentation broth at NIR wavelengths


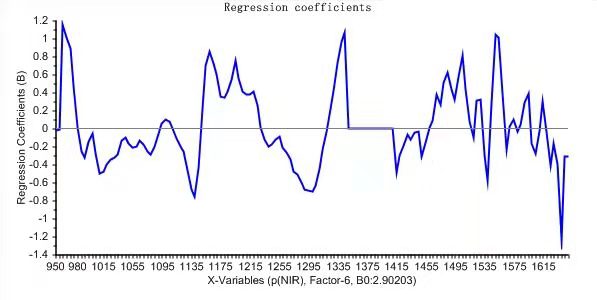


**Fig. S5.** Absorption signals of NH in SG fermentation broth at NIR wavelengths


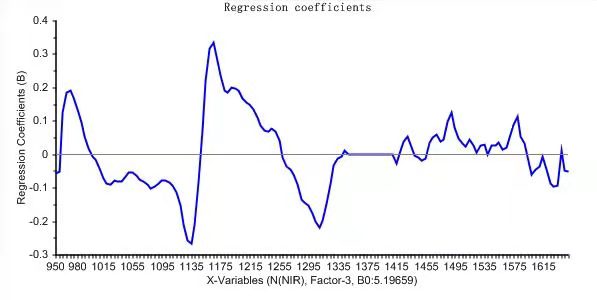


**Fig. S6.** Absorption signals of NH_4_^+^ in SG fermentation broth at NIR wavelengths


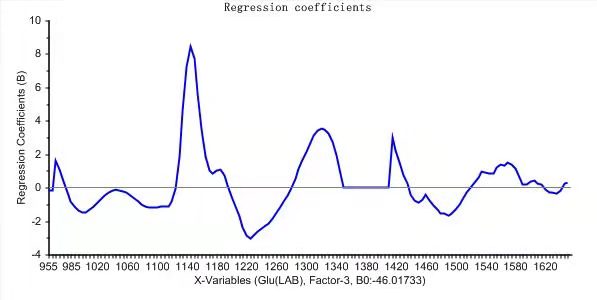


**Fig. S7.** Absorption signals of glucose in SLs fermentation broth at NIR wavelengths


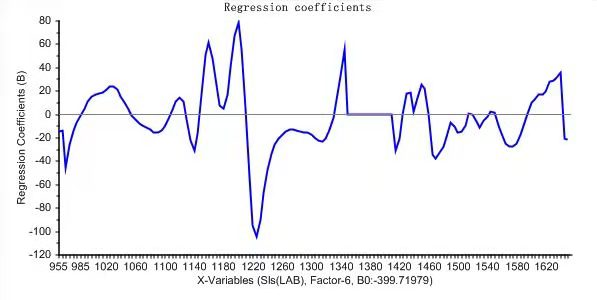


**Fig. S8.** Absorption signals of SLs in SLs fermentation broth at NIR wavelengths


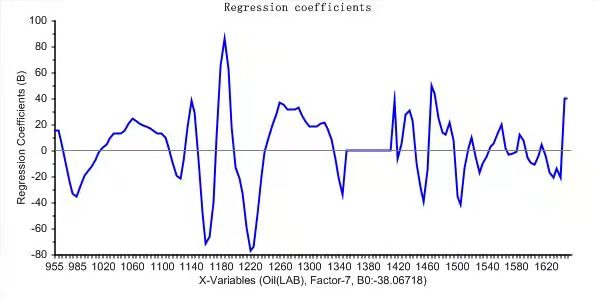


**Fig. S9.** Absorption signals of oil in SLs fermentation broth at NIR wavelengths

*3. validation of model performance index*

**Table S2** Spectral validation of model performance index

|  | L-LA fermentation | | SG fermentation | | | | SLs fermentation | | |
| --- | --- | --- | --- | --- | --- | --- | --- | --- | --- |
|  | Glu | L-LA | Glu | SG | NH_4_^+^ | P | Glu | SLs | Oil |
| RMSEP | 4.023 | 3.351 | 8.014 | 7.012 | 0.100 | 0.0581 | 6.044 | 3.883 | 0.673 |
| R^2^ | 0.996 | 0.990 | 0.985 | 0.975 | 0.985 | 0.986 | 0.988 | 0.996 | 0.994 |
